# Unveiling the Genetic Mosaic of Pediatric AML: Insights from Southwest China

**DOI:** 10.3390/curroncol32110605

**Published:** 2025-10-30

**Authors:** Lan Huang, Xingyu Peng, Wenjing Shu, Hui Shi, Li Xiao, Tao Liu, Yan Xiang, Yuxia Guo, Xianmin Guan, Jiacheng Li, Jie Yu

**Affiliations:** 1 Department of Hematology and Oncology, Children’s Hospital of Chongqing Medical University, Chongqing 400014, China; 2023140305@stu.cqmu.edu.cn (L.H.); 2024111156@stu.cqmu.edu.cn (X.P.); 2022110427@stu.cqmu.edu.cn (W.S.); 2024130299@stu.cqmu.edu.cn (H.S.); 2025140384@stu.cqmu.edu.cn (T.L.); 2023111105@stu.cqmu.edu.cn (Y.X.); 2025140383@stu.cqmu.edu.cn (Y.G.); 2025111289@stu.cqmu.edu.cn (X.G.); 2 National Clinical Research Center for Child Health and Disorders, Ministry of Education Key Laboratory of Child Development and Disorders, Chongqing Key Laboratory of Pediatrics, Chongqing 400014, China; feeceebee@hospital.cqmu.edu.cn; 3 Department of Rehabilitation, Anqing First People’s Hospital of Anhui Medical University, Anqing 246000, China; 4 Big Data Center for Children’s Medical Care, Children’s Hospital of Chongqing Medical University, Chongqing 400014, China; 5 Chongqing Municipal Health Commission Key Laboratory of Children’s Vital Organ Development and Diseases, Chongqing 400014, China

**Keywords:** acute myeloid leukemia, pediatric, transcriptome sequencing, mutations, fusions

## Abstract

**Background:** Pediatric acute myeloid leukemia (pAML) is the second most common type of childhood leukemia, behind acute lymphoblastic leukemia. High-throughput technologies have enabled the identification of increasing molecular alterations linked to AML prognosis, revealing genomic heterogeneity among individual patients and providing clinically valuable diagnostic and prognostic information. This study systematically analyzed the correlation between high-frequency mutated genes and prognosis in pAML by performing whole-transcriptome sequencing (WTS) of bone marrow samples from newly diagnosed AML children in Southwest China and mapping their genetic profiles. **Methods:** pAML patients treated at the Department of Hematology and Oncology, Children’s Hospital of Chongqing Medical University, from January 2015 to October 2024, were enrolled, and WTS was performed. The study described the frequency, pathogenicity classification, and risk stratification of mutation genes and fusion genes, and constructed a genetic landscape. For high-frequency pAML mutations, the impact on early induction remission rate (CR) and long-term event-free survival (EFS) was evaluated. **Results:** A total of 134 pediatric AML patients from Southwest China were included, with a male-to-female ratio of 74:60 and a median diagnosis age of 5.96 years. Based on pathogenicity classification using WTS, fusion genes were categorized into level 1, level 2, and level 3 genes, as well as mutation genes. The study identified five fusion genes of level 1, the most frequent being *RUNX1::RUNX1T1* (32/134, 23.88%), *KMT2A* rearrangements (29/134, 21.64%), and *CBFB::MYH11* (13/134, 9.7%). Sixteen mutation genes of level 1 were detected, seven of which recurred in over 5% of patients, including *NRAS* (31/134, 23.13%), *FLT3* (25/134, 18.66%), *KIT* (24/134, 17.91%), *CEBPA* (14/134, 10.45%), *WT1* (13/134, 9.7%), *KRAS* (11/134, 8.2%), and *PTPN11* (7/134, 5.22%). Sex-based analysis revealed that *PTPN11* mutations were significantly more frequent in males (9.45% vs. 0%, *p* = 0.023), as were *KIT* mutations (24.32% vs. 10.00%, *p* = 0.044). Risk-stratified analysis showed that *WT1* mutations (14.13% vs. 0%, *p* = 0.031) and *FLT3*-ITD mutations (13.19% vs. 0%, *p* = 0.042) were enriched in intermediate- and high-risk groups, whereas *CEBPA* (25.64% vs. 5.43%, *p* = 0.012), *KIT* (35.90% vs. 10.87%, *p* = 0.003), and *KIT*-E8 (20.51% vs. 1.10%, *p* < 0.001) mutations were more prevalent in low-risk groups. Prognostic analysis indicated that *PTPN11* and *KIT* mutations did not affect CR or EFS across sexes, nor did *WT1*, *CEBPA*, or *KIT* mutations influence outcomes by risk stratification. However, *FLT3*-ITD-positive patients had significantly lower CRs (χ^2^ value = 11.965, *p* = 0.007), although EFS differences were nonsignificant. In contrast, *WT1* mutations were associated with inferior EFS compared to wild-type (*p* = 0.036). Furthermore, the univariate and multivariate Cox regression revealed consistent results with the above findings, indicating that *WT1* mutation was an independent adverse prognostic factor for EFS (HR = 2.400, 95% CI: 1.101–5.233, *p* = 0.028). The results of univariate and multivariate logistic regression analyses also confirmed that *FLT3*-ITD mutation was an independent predictor of initial treatment response in our cohort (OR = 10.699, 95% CI: 2.108–54.302, *p* = 0.004). **Conclusions:** This study delineated the genetic landscape of pAML in Southwest China and explored the prognostic value of gene fusions and mutations in early and long-term outcomes. These findings provide a foundation for understanding the genetic heterogeneity of pAML and offer evidence for the development of precision medicine approaches.

## Simple Summary

Acute myeloid leukemia (AML) is the second most common leukemia in children. Modern genetic sequencing helps identify molecular alterations linked to prognosis and reveals genomic heterogeneity among individual patients. This study used whole-transcriptome sequencing to map the genetic landscape of pediatric AML in Southwest China and explored the prognostic value of gene fusions and mutations in early and long-term outcomes. We found that certain gene mutations were more common in males or in specific risk groups. Importantly, *WT1* mutation was an independent risk factor for poorer long-term survival, while *FLT3*-ITD mutation was associated with a lower initial treatment response rate. These findings enhance our understanding of the genetic basis of pediatric AML in this population and may guide more personalized treatment approaches in the future.

## 1. Introduction

Pediatric acute myeloid leukemia (pAML) ranks as the second most prevalent leukemia in children, behind acute lymphoblastic leukemia (ALL) [1]. From 2018 to 2020, the average annual incidence of leukemia was 42.33 per million in children (0–14 years) and 30.08 per million in adolescents (15–19 years) in China [2]. pAML represents 25% of all pediatric acute leukemias. In recent years, outcomes for newly diagnosed pediatric and adolescent AML have improved, with overall survival (OS) rates approaching 65–70% [3]. This progress is largely attributed to the integration of next-generation sequencing (NGS) technology, refinements in intensive chemotherapy regimens, advancements in supportive care, improvements in flow cytometry, and enhanced detection of minimal residual disease (MRD).

NGS has facilitated the identification of numerous molecular alterations associated with AML prognosis, uncovering genomic heterogeneity among individual patients and providing critical diagnostic and prognostic insights [4,5]. The widespread application of NGS has illuminated the genomic landscape of pAML. The most prevalent mutations in pAML include *FLT3*, *NPM1*, *WT1*, *CEBPA*, and *KIT*, while the most common gene fusions involve *RUNX1::RUNX1T1*, *CBFB::MYH11*, and *KMT2A* rearrangements (*KMT2A*-r) [6,7,8]. Recent studies have extensively investigated the prognostic impact of high-frequency mutations and fusion genes in pAML [5,9]. It has been demonstrated that *FLT3*-ITD and *ASXL1* are associated with reduced overall survival, while *CEBPA* and *IDH2* mutations and gene fusions such as *RUNX1::RUNX1T1* and *CBFB::MYH11* correlate with improved overall survival. A study conducted in Shanghai [10] compared the genomic mutation profiles of 292 pAML patients treated at the Shanghai Children’s Medical Center (SCMC) with those in Western pediatric AML cohorts from the TARGET database. This comparison revealed notable differences between the two groups, with Chinese patients exhibiting higher mutation frequencies in *ASXL2*, *JAK2*, *CSF3R* cytoplasmic domain (*CSF3R*-CY) and *KIT* exon 17 (*KIT*-E17), while Western cohorts had elevated mutation frequencies of *FLT3*, *FLT3*-ITD, *NRAS*, *WT1*, *NPM1*, and *TET2*. These findings highlight distinct genomic landscapes across populations with the same clinically defined disease, potentially influenced by genetic or environmental factors. Although this study identified key driver mutations and fusion genes in pAML, its regional focus on Shanghai may introduce potential bias, highlighting the need for data from pAML patients in other regions of China. Genomic research on pAML in China is regionally imbalanced, with studies predominantly concentrated in the developed eastern regions [10], while research in the southwest remains limited. This gap is likely due to the relatively low adoption of NGS technology in hospitals across the southwest. Consequently, establishing a genomic landscape for pAML in Southwest China is essential for advancing regional precision medicine and contributing to the broader genomic database of Chinese pAML.

In the present study, we have established the largest pAML cohort in Southwest China. We performed transcriptome sequencing on a total of 134 newly diagnosed pediatric AML patients and analyzed common mutation genes, fusion genes, and their relationship with prognosis.

## 2. Materials and Methods

### 2.1. Patients and Samples

This study enrolled 134 patients diagnosed with AML at the Department of Hematology and Oncology, Children’s Hospital of Chongqing Medical University, from 2015 to 2024. The inclusion criteria were as follows: (1) aged from 0 to 18 years; (2) diagnosis of AML (excluding acute promyelocytic leukemia/M3); (3) completion of whole-transcriptome sequencing (WTS) at the initial diagnosis of pAML. Patient demographics, clinical characteristics, and outcomes were recorded for analysis. Follow-up data were obtained through outpatient and inpatient electronic medical records, as well as telephone interviews, with the follow-up period concluding in October 2024. The study received ethical approval from the Institutional Review Board of Children’s Hospital, Chongqing Medical University (approval No. 2023.484), and informed consent was obtained from the parents or legal guardians.

### 2.2. Classic Cytogenetic and Fluorescence in Situ Hybridization (FISH) Analysis

Cytogenetic analysis was performed on metaphase cells obtained from bone marrow samples collected before induction chemotherapy using the G-banding technique. Karyotypes were determined according to the International System for Human Cytogenetic Nomenclature (ISCN) [11]. The complex karyotype is characterized by the presence of three or more clonal chromosomal abnormalities, with at least one being a structural aberration [12]. In cases of structural abnormalities, an abnormality was considered clonal if it was present in at least two metaphases. For monosomies, the abnormality was considered significant only if observed in more than three metaphases.

Based on the karyotyping results, fluorescence in situ hybridization (FISH) was performed on interphase nuclei using commercially available probes to further confirm specific chromosomal translocations and detect microdeletions [13]. The AML panel included dual-color dual-fusion probes for t(8;21) (*RUNX1::RUNX1T1*) and t(15;17) (*PML::RARA*), as well as dual-color break-apart probes for *KMT2A* (11q23) and inv(16)/t(16;16) (*CBFB::MYH11*). Two hundred unstimulated interphase cells were analyzed for each probe, and the results were reported according to the standards provided by ISCN.

### 2.3. Risk Stratification of pAML Patients

At the time of diagnosis, patients were stratified into three risk groups based on cytogenetics and molecular abnormality, white blood cell (WBC) count, and central nervous system (CNS) infiltration. Cytogenetic and molecular risk stratification was as follows: low-risk (LR) patients met one or more of the following criteria: t(8;21)-*AML1::ETO*, inv(16) or t(16;16)-*CBFβ::MYH11*, *CEBPA* mutation without *FLT3*-ITD, *NPM1/IDH* mutation without *FLT3*-ITD. High-risk (HR) patients met any of these criteria: t(6;9), t(8;16), t(6;21), −7, −5, inv(3)/t(3;3)-*RPN1::EVI1*, *FLT3*-ITD mutation, therapy-related AML, or MDS-related AML. Patients who did not meet criteria for either LR or HR groups were classified as intermediate-risk (IR) (Supplementary Figure S1A). Risk stratification was reassessed following induction therapy. LR pAML patients failing to achieve complete remission (CR) were reclassified as IR, while IR patients who did not achieve CR were upgraded to HR. HR patients failing to achieve CR received alternative, hematologist-guided regimens.

### 2.4. Treatment Protocol

The chemotherapy treatment protocol and dosing regimens are detailed in Supplementary Figure S1B. According to the pAML treatment protocol, therapy consists of four courses across two phases. In the first course of induction therapy, the ADE (10 + 3 + 5) regimen was administered. Following risk stratification, chemotherapy regimens were adjusted based on the patient’s risk category for the second course of induction therapy. Patients in the LR group received the ADE (8 + 3 + 5) regimen, while those in intermediate or HR groups received the AM regimen. After induction therapy, bone marrow (BM) examination was performed for risk reassessment. Patients achieving CR proceeded to intensification therapy, while non-responders entered alternative research protocols. During the first course of intensification therapy, the AE regimen was used. In the second course, LR patients received the AM regimen, while intermediate or HR patients with an available donor underwent allogeneic hematopoietic stem cell transplantation (HSCT) after the first intensification course. Those without a suitable donor were treated with AL regimens.

### 2.5. Definition and Outcome

Central nervous system leukemia (CNSL) is an extramedullary leukemia caused by the infiltration of leukemia cells into the meninges, cranial nerves, brain tissue, and spinal cord [14]. The primary endpoints of this study included CR and event-free survival (EFS). CR was defined as the presence of <5% blast cells in BM [15], with patients considered to have achieved CR after assessment following the second course of induction therapy. EFS was defined as the time from diagnosis to the first major adverse event, including failure to achieve remission, relapse after remission, death from any cause, or the development of a secondary malignancy.

### 2.6. Transcriptome Sequencing (RNA-Seq) and Analysis

Total RNA was extracted from the mononuclear cells of BM samples of AML patients. Then, RNA concentration, purity, and integrity were assessed prior to library construction. The library was constructed using 25−1000 ng of RNA from each sample, following quality evaluation. Library concentration and the fragment size were determined using the Agilent 2100 Bioanalyzer instrument. The constructed RNA library was sequenced using the Illumina HiSeq X platform in 150 PE mode, with a minimum sequencing depth of 15 G PF data per sample. Variant detection was performed using VarScan, while gene fusion prediction was carried out with Arriba. Mutation results were annotated using ANNOVAR (version 2020−06−07) for downstream data analysis, with annotation databases including ClinVar, dbSNP, 1000 Genomes, gnomAD, ExAC, COSMIC, and others. Gene fusion databases, including but not limited to COSMIC, Fusion Cancer, Atlas of Genetics and Cytogenetics in Oncology and Hematology, and My Cancer Genome, were also consulted.

### 2.7. Classification Criteria for Level 1–3 Fusion Genes and Mutated Genes

In accordance with the Standards and Guidelines issued by the Association for Molecular Pathology (AMP), American Society of Clinical Oncology (ASCO), and College of American Pathologists (CAP), and combined with the 2018 Chinese Expert Consensus on NGS Applications in Hematologic Malignancies, fusion genes and mutated genes were classified into three levels (1−3) [16] (Supplementary Table S1).

### 2.8. Statistical Analysis

**Figure 1:**
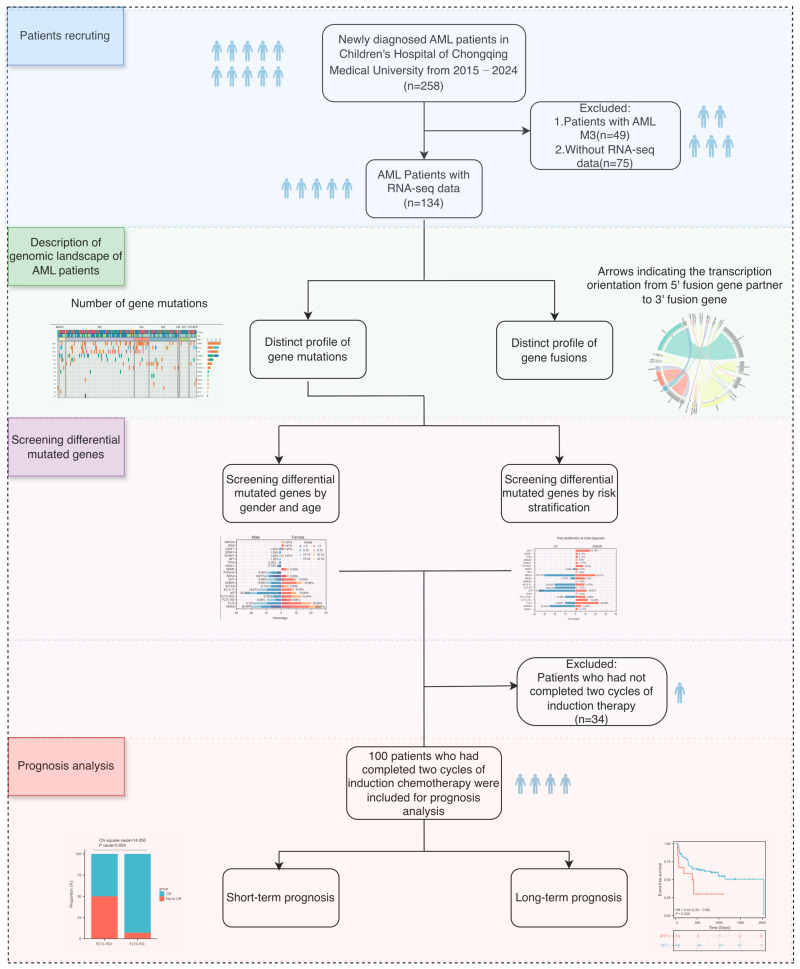
The workflow chart of the study. AML: acute myeloid leukemia; RNA-seq: Transcriptome sequencing; CR: complete remission; To represent the cohort size more clearly, a human symbol was used to denote approximately 25 pediatric patients.

A flow diagram of this study is presented in Figure 1. Differences in mutation frequencies among both sexes and different risk stratifications were assessed using Fisher’s exact test or Chi-square test, as appropriate. Multiple comparisons were conducted by Bonferroni’s Multiple Comparison Test. For prognosis of pAML, CR rates were compared using the Chi-square test or Fisher’s exact test. Survival analysis was conducted using the Kaplan–Meier estimation to evaluate the prognostic value of genetic variants. The Coxregression model was used to evaluate the impact of clinical characteristics and genetic variations on prognosis, and the logistic regression model was used to assess the influence of clinical characteristics and genetic variations on CR. The analysis was performed using SPSS (version 27.0), GraphPad Prism (version 10.0), and R Studio (version 4.1.0). All the tests were two-tailed with a significance level of 0.05.

## 3. Results

### 3.1. Clinical Characteristics

This study enrolled a total of 134 pAML patients, comprising 74 males and 60 females. The clinical characteristics of these patients are presented in Table 1. The median age was 5.96 (3.42, 11.48) years old. The median WBC count was 13.88 (5.55, 46.79) × 10^9^/L.

RNA sequencing was performed on all BM samples included in the study. Mutated and fusion genes were categorized into pathogenicity levels 1–3 (Supplementary Table S1). A total of 555 mutated genes with distinct pathogenicity levels were identified, with 16 (16/555, 2.88%) classified as level 1, 58 (58/555, 10.45%) as level 2, and 481 (481/555, 86.67%) as level 3. Additionally, 67 fusion genes were identified, including 5 (5/67, 7.46%) classified as level 1, 8 (8/67, 11.94%) as level 2, and 54 (54/67, 80.60%) as level 3. Karyotype analysis revealed that 25.37% of the patients (34 out of 134) exhibited a normal karyotype, whereas 66.42% (89 out of 134) showed abnormal karyotypes with diverse or complex chromosomal abnormalities, and 8.21% (11 out of 134) had an unknown karyotype. Among patients with abnormal karyotypes, t(8;21)(q22;q22) was the most common structural chromosomal abnormality, observed in 30 patients (30/89, 33.71%). A total of 21 patients underwent HSCT, with the majority classified as FAB-M2 (9/21, 42.86%) and FAB-M5 (9/21, 42.86%). In this subgroup, the most prevalent fusion gene was *KMT2A* rearrangement (*KMT2A*-r) (7/13, 53.85%), and the most frequent mutated gene was *WT1* (5/22, 22.73%) (Supplementary Table S2).

### 3.2. Gene Fusions Identified in Pediatric AML Patients

**Figure 2:**
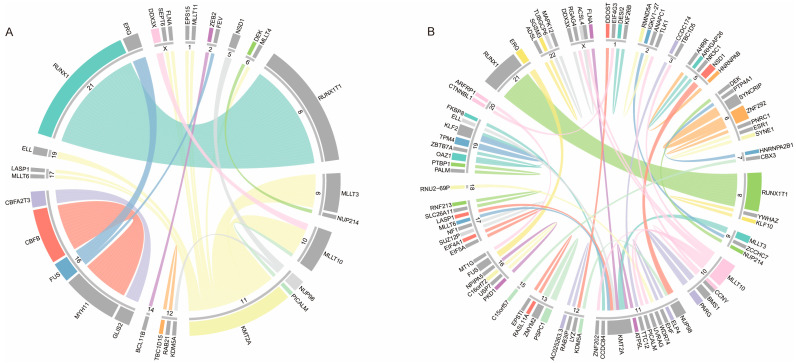
Overview of all gene fusions detected in the pediatric AML cohort. The circos plot shows level 1–2 (**A**) and level 3 (**B**) fusion genes identified in pediatric AML patients, illustrating the chromosomal origin of the fusion genes. Arrows indicate the transcription orientation from the 5′ fusion gene partner to the 3′ fusion gene. Fusion genes with multiple partners are grouped and color-coded, and the partner gene at the 3′ end is gray.

RNA-seq data analysis revealed that 97 out of 134 patients (72.39%) exhibited 162 rearrangements involving 102 genes (Figure 2, Table 2, and Supplementary Table S3). Figure 2 displays the identified level 1–3 fusion genes, with both ends of the connecting lines anchored to the chromosomal coordinates of the partner genes, representing the joining of two breakpoints in the fusion gene. The highest-frequency fusion events in Southwestern Chinese pediatric AML cohorts were *RUNX1::RUNX1T1* (32/134, 23.88%), *KMT2Ar* (29/134, 21.64%), and *CBFB::MYH11* (13/134, 9.70%). However, we identified only 3 cases of *NUP98* rearrangements, including *NUP98::KDM5A* (1/134, 0.75%) and *NUP98::NSD1* (2/134, 1.49%), which differs from the previously reported high incidence of *NUP98* rearrangements in pAML patients [10,17]. Interestingly, we identified four patients with the *CBFA2T3::GLIS2* fusion gene, all of whom were classified as FAB-M7 subtype with a notably young median age of 1.83 years, consistent with previous literature reports [18]. Additionally, all patients with the *RUNX1::RUNX1T1* fusion gene were classified as FAB-M2, while the majority of patients with *KMT2A*-r belonged to the FAB-M5 group (20/29, 68.97%).

### 3.3. Gene Mutation Landscape in Pediatric AML Patients

#### 3.3.1. Summary Mutation Profile

**Figure 3:**
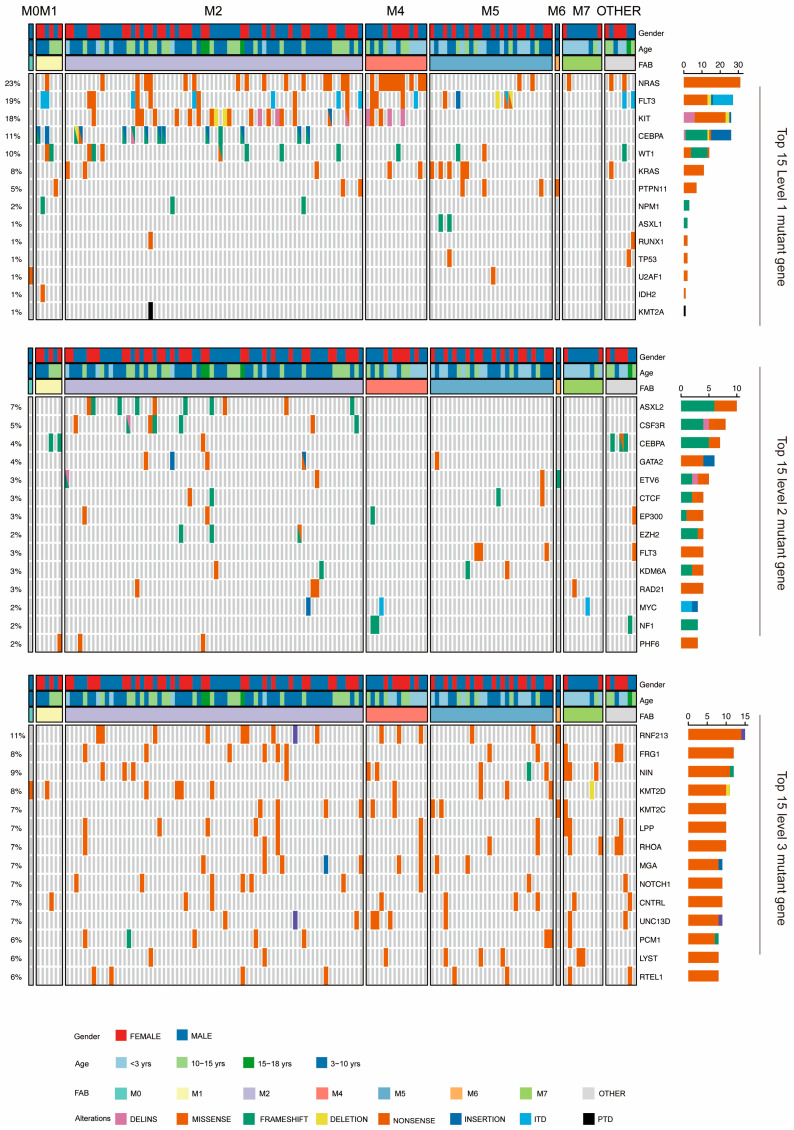
Landscape of mutated genes in the pediatric AML cohort. The heatmap displays the top 15 level 1–3 mutated genes. Cases are divided into FAB groups (columns). Color represents different mutation types. The percentages on the left side of the heatmap indicate the mutation frequency, while the numbers on the right represent the mutation count. Note: The same gene may be classified as different levels of mutated genes due to varying pathogenicity at different mutation sites. For example, *CEBPA* is present in both level 1 and level 2 mutated gene lists.

The mutation landscape outlines the frequency and types of mutations in level 1–3 genes (Figure 3). Among the level 1 mutated genes, seven were recurrently mutated in over 5% of patients, including *NRAS* (31/134, 23.13%), *FLT3* (25/134, 18.66%), *KIT* (24/134, 17.91%), *CEBPA* (14/134, 10.45%), *WT1* (13/134, 9.7%), *KRAS* (11/134, 8.2%), and *PTPN11* (7/134, 5.22%) (Table 3). Among them, the incidence rates of *FLT3*-ITD, *FLT3*-TKD, *KIT* exon 17 (*KIT*-E17), and *KIT* exon 8 (*KIT*-E8) mutations in the cohort were 8.96%, 10.45%, 11.94%, and 6.72%, respectively. Notably, *NRAS*, *KRAS*, and *PTPN11* mutations were predominantly missense mutations. The classification of mutated genes into different levels was based on their established pathogenic potential (Supplementary Table S1). Among level 2 mutations, which are considered driver mutations pending further validation, the most frequently observed were *ASXL2* (10/134, 7.46%), *CSF3R* (7/134, 5.22%), *CEBPA* (6/134, 4.48%), *GATA2* (5/134, 3.73%), and *EP300* (4/134, 2.99%). Level 3 mutations, classified as uncertain significance, were dominated by *RNF213* (15/134, 11.19%), *NIN* (12/134, 8.96%), *FRG1* (11/134, 8.21%), *KMT2D* (11/134, 8.21%), and *KMT2C* (10/134, 7.46%). Notably, the high frequency of epigenetic regulators (*KMT2D* and *KMT2C*) in the level 3 group suggested their potential role in disease pathogenesis that warrants further investigation, despite their current classification as variants of uncertain significance. This level distribution provides a framework for prioritizing molecular findings while acknowledging that the clinical and biological significance of some mutations may evolve with additional evidence.

#### 3.3.2. Co-Mutation Profiles in Pediatric AML Patients

**Figure 4:**
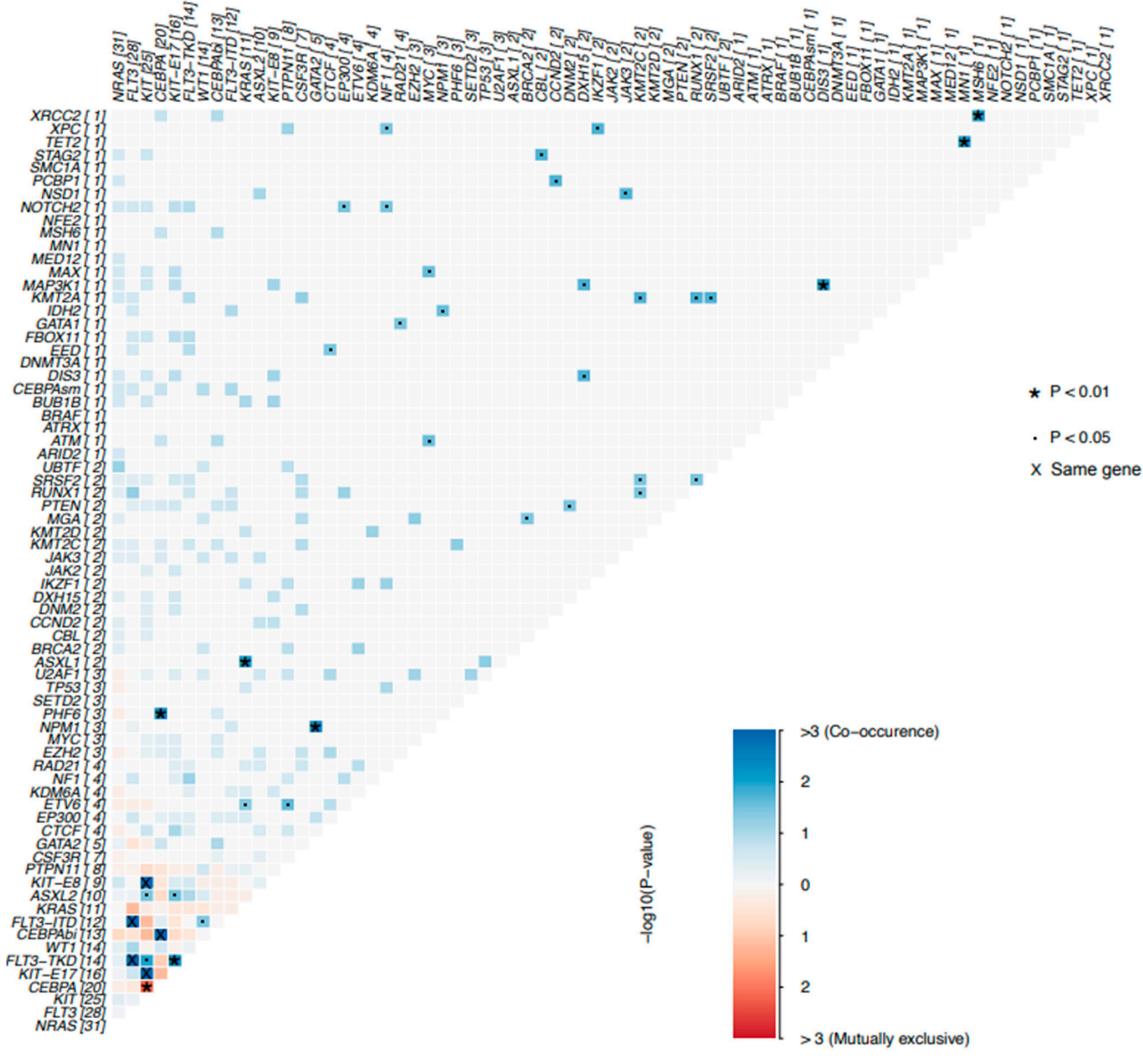
Co-mutations and exclusivity patterns of the AML cohort. (two-sided Fisher’s exact test). Blue: co-occurrence relationship; Red: exclusivity relationship. Darker colors indicate stronger associations.

Furthermore, we investigated the pairwise relationships of levels 1–2 mutated genes detected in our cohort. A total of 71 pairs of mutated genes were analyzed for co-mutation. Our analysis revealed several significant co-mutation pairs in pAML, such as *KIT*-E17 and *FLT3*-TKD (*p* = 0.003), *PHF6* and *CEBPA* (*p* = 0.004), *NPM1* and *GATA2* (*p* = 0.004), *ASXL1* and *KRAS* (*p* = 0.008), *ASXL2* and *KIT*-E17 (*p* = 0.029), *ETV6* and *PTPN11* (*p* = 0.023), among others (Figure 4 and Supplementary Table S4).

#### 3.3.3. Sex-Related and Risk-Stratification-Related Mutational Profile

**Figure 5:**
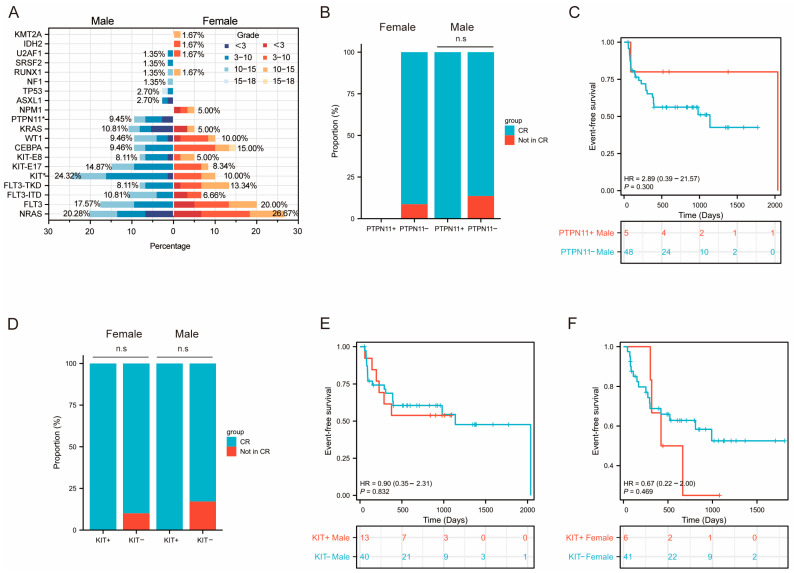
Mutational profile in age and gender. (**A**) Differing level 1 mutation recurrence between males and females. Left (blue), mutation recurrences in males. Right (red), mutation recurrences in females. Patients were categorized into different age groups: <3 years, 3–10 years, 10−15 years, and 15−18 years (with varying color intensity representing different age groups). Asterisks indicate statistical significance. (*p* < 0.05, two-sided Fisher’s exact test). (**B**) The bar plot shows the proportion of patients achieving CR among *PTPN11*-positive and *PTPN11*-negative cases, respectively, in males and females. (**C**) Kaplan–Meier curves of event-free survival of patients with and without *PTPN11* mutation in males. (**D**) The bar plot shows the proportion of patients achieving CR among *KIT*-positive and *KIT*-negative cases, respectively, in males and females. Kaplan–Meier curves of event-free survival of patients with and without *KIT* mutation in males (**E**) and females (**F**).

Subsequently, we focused our analysis on level 1 mutated genes. The RNA-seq results revealed distinct mutation profiles based on sex. *PTPN11* mutations occurred more frequently in male patients than in females (9.45% vs. 0% in males and females, respectively, *p* = 0.023), and *KIT* mutations showed a similarly elevated prevalence in males (24.32% vs. 10.00%, *p* = 0.044) (Figure 5A). Further analysis of the age distribution of level 1 mutations revealed a lower proportion of older children (15−18 years) among those with level 1 mutations, likely due to this age group’s tendency to seek treatment at adult hospitals. Moreover, several key mutations showed uneven age distributions: *KRAS* mutations (6/11, 54.55%) were most common in children under 3 years, while *CEBPA* (68.75%, 11/16) and *KIT* (62.50%, 15/24) mutations peaked in early childhood (3−10 years). In contrast, *WT1* (15.38%, 2/13) and *FLT3* (12%, 3/25) mutations were least common in the <3 years age group.

**Figure 6:**
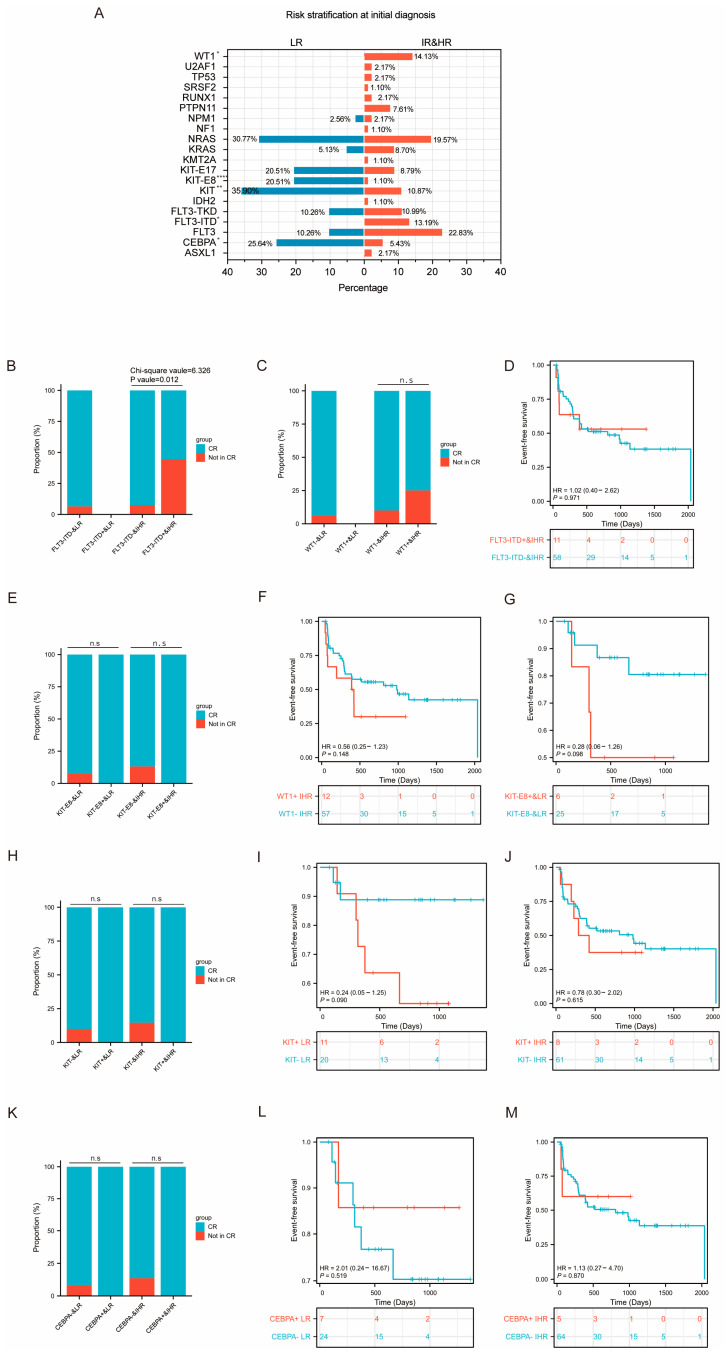
The mutation landscape in different risk groups. (**A**) Differing level 1 mutation recurrence between the low-risk group and the intermediate- and high-risk groups. Left (blue), mutation recurrences in the low-risk group. Right (red), mutation recurrences in the intermediate- and high-risk group. Asterisks indicate statistical significance. (* *p < 0.05*, ** p < 0.01, **** p < 0.0001. two-sided Fisher’s exact test). (**B**) The bar plot shows the proportion of patients achieving CR among *FLT3-ITD*-positive and *FLT3-ITD*-negative cases in the intermediate- and high-risk groups. (**C**) The bar plot shows the proportion of patients achieving CR among *WT1*-positive and *WT1*-negative cases, respectively, in different risk groups. (**D**) Kaplan–Meier curves of event-free survival of patients with and without *FLT3-ITD* mutation in the intermediate- and high-risk groups. (**E**) The bar plot shows the proportion of patients achieving CR among *KIT*-E8 positive and *KIT*-E8 negative cases, respectively, in different risk groups. (**F**) Kaplan–Meier curves of event-free survival of patients with and without *WT1* mutation in the intermediate- and high-risk groups. (**G**) Kaplan–Meier curves of event-free survival of patients with and without *KIT*-E8 mutation in the low-risk group. (**H**) The bar plot shows the proportion of patients achieving CR among *KIT*-positive and *KIT*-negative cases, respectively, in different risk groups. Kaplan–Meier curves of event-free survival of patients with and without *KIT* mutation in the low-risk group (**I**) and the intermediate- and high-risk groups (**J**). (**K**) The bar plot shows the proportion of patients achieving CR among *CEBPA*-positive and *CEBPA*-negative cases, respectively, in different risk groups. Kaplan–Meier curves of event-free survival of patients with and without *CEBPA* mutation in the low-risk group (**L**) and the intermediate- and high-risk groups (**M**).

The mutation landscape also illustrated mutation distributions across different risk groups (Figure 6A). Mutations in *KIT* (35.90% vs. 10.87% in the low-risk group and the intermediate- and high-risk groups, respectively, *p* = 0.003), *KIT*-E8 (20.51% vs. 1.10%, *p* < 0.001) and *CEBPA* (25.64% vs. 5.43%, *p* = 0.012) were found to be overrepresented in the low-risk group, while patients in the intermediate- and high-risk groups had a higher frequency of mutations in *WT1* (14.13% vs. 0%, *p* = 0.031) and *FLT3*-ITD (13.19% vs. 0%, *p* = 0.042).

#### 3.3.4. Prognosis Analysis of Differentially Mutated Genes

Comprehensive prognostic analyses of *PTPN11*, *KIT*, *KIT*-E8, *WT1*, *FLT3*-ITD and *CEBPA* mutations were performed in the cohort, stratified by both sex and risk stratification groups. Initial sex-specific comparisons revealed that while *PTPN11* and *KIT* mutations showed significant gender disparities in prevalence, neither mutation significantly impacted clinical outcomes in either sex. In male patients, no significant differences in CR rates or EFS were observed between those with and without PTPN11 or KIT mutations. Similarly, in female patients, KIT-mutated cases exhibited CR rates and EFS outcomes comparable to wild-type controls (Figure 5B−F). Subsequent risk-stratified analysis demonstrated that in the intermediate- and high-risk groups, patients with *FLT3*-ITD mutations were less likely to achieve complete remission (CR) compared to those without the mutation (χ^2^ value =6.326, *p* value = 0.012) (Figure 6B); no difference in EFS was observed (Figure 6D). However, the results showed no significant prognostic effect of *WT1*, *KIT*, *KIT*-E8, or *CEBPA* mutations across risk groups (Figure 6C,E−M). In low-risk patients, neither *KIT* nor *CEBPA* mutations affected outcomes compared to wild-type. Intermediate- and high-risk patients with *WT1*, *KIT*, or *CEBPA* mutations likewise showed equivalent CR rates and EFS to wild-type counterparts. These findings suggested that not all frequently occurring gene mutations carried equal prognostic weight. Although *WT1*, *CEBPA*, *KIT*, and *KIT*-E8 mutations exhibited distinct distribution patterns, they may not independently drive clinical outcome differences in this population. In contrast, *FLT3*-ITD emerged as a strong independent adverse prognostic factor in intermediate- and high-risk groups, directly contributing to leukemia cell resistance to initial induction chemotherapy.

### 3.4. Prognosis Analysis of Level 1 Mutated Genes

**Figure 7:**
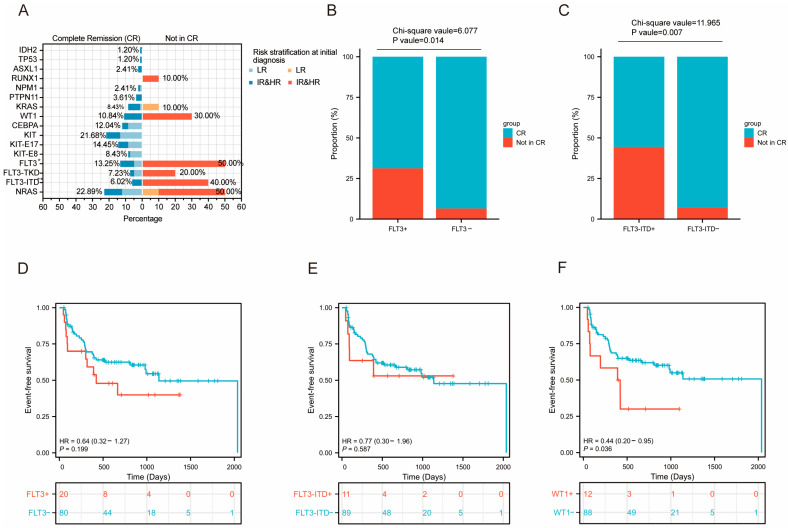
Prognosis analysis of level 1 mutated genes. (**A**) The proportion of patients carrying level 1 mutated genes achieving CR versus those not achieving CR. Left (blue), the proportion of patients carrying level 1 mutated genes who achieved CR. Right (red), the proportion of patients carrying level 1 mutated genes who did not achieve CR. Patients were categorized into different risk groups: the low-risk group (light color) and the intermediate- and high-risk groups (dark color). Asterisks indicate statistical significance. ((* p < 0.05, ** p < 0.01, two-sided Fisher’s exact test). (**B**) The bar plot shows the proportion of patients achieving CR among *FLT3*-positive and *FLT3*-negative cases. (**C**) The bar plot shows the proportion of patients achieving CR among *FLT3-ITD*-positive and *FLT3-ITD*-negative cases. (**D**) Kaplan–Meier curves of event-free survival of patients with and without *FLT3* mutation. (**E**) Kaplan–Meier curves of event-free survival of patients with and without *FLT3-ITD* mutation. (**F**) Kaplan–Meier curves of event-free survival of patients with and without *WT1* mutation (*p* = 0.036).

Prognostic analysis was performed on level 1 mutated genes with a mutation frequency exceeding 5% in the cohort. The results revealed that patients with *FLT3* (χ^2^ value =6.077, *p* value = 0.014) or *FLT3*-ITD (χ^2^ value =11.965, *p* value = 0.007) mutations were less likely to achieve CR compared to those without the mutation, although no significant difference in EFS was observed (Figure 7A−E), These results suggest that *FLT3* and *FLT3*-ITD mutations negatively impacted early treatment response, but did not influence long-term outcomes. *WT1*-mutated patients showed comparable CR rates. However, Kaplan–Meier analysis demonstrated that patients with *WT1* mutations had significantly inferior EFS compared to those with the wild-type genotype (*p* value = 0.036; Figure 7F). Subsequently, to evaluate the independent prognostic value of *WT1* for EFS, we conducted univariate and multivariate Cox regression analyses (Supplementary Table S5). Variables with a *p*-value < 0.1 in the univariate analysis were then included in the multivariate analysis. The multivariate regression analysis revealed that *WT1* mutation was independently associated with poor prognosis (HR = 2.400, 95% CI: 1.101−5.233, *p* = 0.028). Additionally, to investigate factors influencing CR, we performed univariate and multivariate logistic regression analyses (Supplementary Table S6). The results of the multivariate logistic regression analysis demonstrated that *FLT3*-ITD was an independent negative predictor of achieving CR (OR = 10.400, 95% CI: 2.196−49.247, *p* = 0.003). Furthermore, no significant differences in either short-term (CR) or long-term (EFS) outcomes were observed for patients with *CEBPA*, *KIT*, *PTPN11*, *KRAS*, or *NRAS* mutations compared to patients without mutations (Supplementary Figure S2A−H).

## 4. Discussion

Advancements in genomic technologies, particularly NGS, have enhanced our comprehension of the genetic landscape of AML, leading to the identification of numerous mutations and fusions that could serve as prognostic markers for AML.

In this study, we identified *RUNX1::RUNX1T1*, *KMT2A*-r, and *CBFB::MYH11* as the most prevalent fusion genes. However, the incidence of *NUP98* rearrangements in our cohort was relatively low (3/134, 2.24%), which contrasted with previous reports reporting higher frequencies in pediatric AML. For instance, *NUP98* rearrangements were the third most frequently occurring fusion gene in the Shanghai SCMC cohort, with an incidence rate as high as 5.8% [10], while the COG trial cohort showed a 7.2% occurrence rate [17]. One potential explanation could be the differences in genetic backgrounds across different regions.

In our cohort, the most frequently mutated genes included *NRAS*, *FLT3*, *KIT*, *CEBPA*, and *WT1*. The mutational profile of our cohort differed significantly from those of both the Shanghai SCMC cohort and pediatric AML cohorts from the Western TARGET database [10]. Specifically, *NRAS* was the most common mutation in our cohort (23.13%), contrasting with *FLT3* in Western cohorts (31.7%). Additionally, mutations in RAS pathway genes, such as *NRAS* (31.5% vs. 23.13%) and *KRAS* (14.3% vs. 8.2%), were significantly more prevalent in Western cohorts than in ours [19]. It is also noteworthy that the *NPM1* mutation frequency was markedly higher in Western cohorts (8.6% vs. 2.24%) than in our cohort [20]. When comparing our cohort with the Shanghai SCMC cohort, we observed differences in the prevalence of key gene mutations. For example, *WT1* mutations were more frequent in our cohort (9.70%) than in the SCMC cohort (2.4%). While *WT1* mutation did not impact prognosis in the SCMC cohort, it emerged as an independent risk factor in our Southwest China cohort (HR = 2.400, 95% CI: 1.101−5.233, *p* = 0.028). This finding suggested that pediatric AML patients with *WT1* mutations in our regional population may require closer monitoring and more tailored treatment strategies. Our results highlight the notable ethnic and regional heterogeneity in the genomic landscape of pediatric AML. Consequently, directly applying epidemiological data and risk stratification models derived from Western populations to Chinese pediatric patients is inadequate. This study provides essential insights for developing population-specific risk models that better reflect the genetic background and clinical characteristics of Chinese children with AML, particularly by accounting for ethnic and geographic variations. Furthermore, compared to adult AML, distinct mutational spectra were observed. Common mutations in adult AML typically include *TET2*, *ASXL1*, *GATA2*, *TP53*, and *DNMT3A*, reflecting age-related differences in mutation patterns [21,22]. A contributing factor may be the high proliferative state of leukemia stem cells (LSCs) in pAML, with activated RAS signaling pathways [23], which could explain the elevated mutation frequencies in RAS pathway genes such as *NRAS*, *KRAS*, and *PTPN11* [24]. In contrast, adult AML LSCs tend to enter a more quiescent state, creating selective pressure that favors the acquisition of mutations in epigenetic regulators such as *TET2* and *DNMT3A* [25]. These findings indicated the necessity of incorporating ethnic and regional genetic backgrounds when analyzing AML genomic data, highlighting the importance of adapting risk stratification approaches and therapeutic strategies according to distinct population characteristics.

HSCT holds significant value for the prognosis of pAML. Particularly, evaluating the outcomes of pAML patients with different fusion genes and mutant genes following HSCT treatment could provide crucial insights for clinical decision-making. However, in this study, only 21 pAML patients completed HSCT treatment, and the number of cases involving specific fusion genes and mutant genes was insufficient for statistical analysis. Therefore, an in-depth discussion was not feasible.

In our cohort, four cases with the *CBFA2T3::GLIS2* fusion gene were identified, all of which were classified as FAB-M7 subtype. The *CBFA2T3* gene, also known as *MTG16* or *ETO2*, is a transcriptional repressor. Previous studies have identified *CBFA2T3* as a fusion partner of *RUNX1* in AML [26]. *GLIS2*, a transcription factor, involved in kidney development, is not normally expressed in the hematopoietic system. The frequent occurrence of the *CBFA2T3::GLIS2* fusion in M7-type leukemia may be attributed to the enhanced BMP (bone morphogenetic protein) signaling which may directly contribute to megakaryocytic differentiation in leukemia cells [18]. Interestingly, we also identified four cases with *ZNF292* rearrangements, including three *ZNF292::SYNCRIP* fusions and one *ZNF292::PNRC1* fusion. Notably, *ZNF292* rearrangements have not been previously reported in AML. The *ZNF292* gene is a tumor suppressor gene, and mutations in this gene have been reported to be associated with the development of gastric cancer, colorectal cancer, and leukemia [27,28]. *SYNCRIP* is an RNA-binding protein critical for maintaining self-renewal and proteostasis in hematopoietic stem and progenitor cells [29]. Previous studies have also identified *SYNCRIP* as a critical RNA-binding protein that regulates the leukemogenic transcriptional program in myeloid leukemia [30]. Additionally, *SYNCRIP* has been reported to be involved in the tumorigenesis of prostate cancer and lung cancer [31,32]. On the other hand, a study by Gaviraghi et al. [33] showed that *PNRC1* exerted tumor-suppressive effects by regulating ribosome biogenesis. Further experiments are needed to elucidate the function of this fusion gene. Our analysis revealed several novel gene rearrangements in this cohort, such as *TUBGCP6::MAPK12* (*n* = 1), *SUZ12P::NF1* (*n* = 1), and *SGSM3::ADSL* (*n* = 1), which have not been previously observed (Supplementary Table S7). These newly identified fusion genes warrant further investigation to explore their functional roles in leukemogenesis.

Specific mutations are closely associated with distinct patient characteristics such as gender and age, which have significant implications for investigating pathogenesis, guiding treatment strategies, and predicting prognosis. Our study identified several gender-associated mutated genes, with *KIT* and *PTPN11* mutations being more prevalent in male patients, consistent with prior findings [34]. Notably, both *KIT* and *PTPN11* are located on autosomes. To our knowledge, this is the first report demonstrating a male bias for *KIT* and *PTPN11* mutations in pAML. A study of 1726 adult AML cases [34] similarly found a higher frequency of *KIT* mutations in males. Additionally, PTPN11 mutations exhibit a male predilection in Noonan syndrome [35]. However, the underlying mechanisms for this gender bias in mutational prevalence remain unclear. Large-scale genomic studies have identified sex-biased gene regulatory networks that may contribute to these gender disparities in mutation frequencies [36]. Subsequent prognostic analysis of *KIT* and *PTPN11* mutations showed that neither mutation significantly affected clinical outcomes in either gender. This observation suggested that *KIT* and *PTPN11* may not function as key prognostic driver genes independently, but rather require cooperative interactions with co-occurring mutations to determine patient outcomes. For instance, *KIT* frequently co-mutates with *ASXL2* and *CSF3R*. Therefore, further investigation is warranted to elucidate the prognostic implications of these mutational cooperativities in pAML. Furthermore, our study revealed distinct age-stratified mutational profiles in pAML. *KRAS* mutations were notably more frequent in infants under 3 years old, while *CEBPA* and *KIT* mutations were predominantly enriched in children aged 3–10 years. These differential mutational patterns reflect developmental stage-specific transformation mechanisms in hematopoietic stem cells [37] and provide important insights for precision diagnosis and tailored therapeutic approaches. This study also found that mutations in *KIT*, *KIT*-E*8* and *CEBPA* were overrepresented in the low-risk group, while patients in the intermediate- and high-risk groups had a higher frequency of mutations in *WT1* and *FLT3*-ITD. According to current molecular genetic risk stratification criteria, patients with *CEBPA* mutations in the absence of other high-risk factors can be classified into the low-risk group and patients with *FLT3*-ITD mutations can be classified into the high-risk group. However, *KIT*-E8 and *WT1* mutations have not yet been incorporated into the risk stratification system, suggesting potential for optimization of current standards. This study recommends that future updates to risk stratification guidelines consider formally including *WT1* mutations as a high-risk factor, while simultaneously conducting a more refined reassessment of the prognostic significance of *KIT*-E8 mutations.

To explore the role of gene co-mutations in the development and progression of AML, we also investigated the pairwise relationship of mutations. The associations between *ASXL2* and *KIT*, and between *NPM1* and *GATA2*, were also previously identified in the SCMC cohort [10]. However, different mutation associations were observed across different populations. For example, in Western populations, *NPM1* mutations often co-occur with *FLT3*-ITD mutations [38], while *CSF3R* mutations co-occur with *CEBPA* mutations [7].

Our findings demonstrated that patients with *FLT3*-ITD mutation were significantly less likely to achieve CR compared to patients without; however, there was no difference in EFS compared to patients without *FLT3*-ITD mutations. This indicates that the FLT3-ITD mutation directly contributes to leukemia cell resistance to initial induction chemotherapy. The *FLT3*-ITD mutation leads to constitutive activation of downstream signaling pathways, such as STAT5 and MAPK [39], promoting excessive proliferation of cancer cells and conferring resistance to chemotherapy-induced apoptosis. The discrepancy between the significant negative impact of *FLT3*-ITD on the CR rate and its non-significant effect on EFS may be attributable to post-induction therapeutic strategies. According to the treatment protocol (Supplementary Figure S1B), patients who did not achieve CR after induction chemotherapy were up-classified into a higher-risk group and subsequently received more intensive chemotherapy regimens. This timely adjustment in treatment strategy effectively cleared residual leukemic cells, enabling some initially resistant patients to achieve remission ultimately. It is plausible that these subsequent interventions attenuate, or even abrogate, the initial negative impact of the mutation, thereby minimizing differences in EFS. Our study also revealed that *WT1*-mutated patients had comparable CR rates but inferior EFS relative to *WT1*-wild-type patients, which were consistent with previous studies [40]. The *WT1* mutations may disrupt normal hematopoietic differentiation, conferring leukemia cells enhanced self-renewal capacity and survival advantages [41,42]. This significantly increases the risk of relapse from persistent MRD. Therefore, both *FLT3*-ITD and *WT1* should be regarded as risk markers for AML. For *FLT3*-ITD-positive patients, the results strongly support the use of *FLT3* inhibitors such as Gilteritinib during induction chemotherapy or after remission to overcome initial drug resistance. Additionally, *WT1* mutation sequences can serve as an excellent quantifiable MRD monitoring marker [43], allowing for close surveillance for signs of relapse during the remission phase. Based on our findings, routine mutation screening for *WT1* and *FLT3*-ITD genes at diagnosis is recommended for comprehensive risk stratification and to guide chemotherapy regimen selection and targeted therapy decisions.

## 5. Conclusions

In summary, this study provides a comprehensive analysis of the genetic landscape of pAML in southwestern China and elucidates the prognostic implications of these genetic variations. The findings not only lay the foundation for further investigation into the role of mutant genes in pAML pathogenesis and progression, but also highlight the importance of considering ethnic and geographic variations when establishing risk-stratified clinical management strategies in the era of precision medicine.

## Figures and Tables

**Table 1:** Baseline Characteristics of the Pediatric AML Cohort.

Indicators	N (%)	Indicators	N (%)
Gender		AGE	
Female	60 (44.78)	P50 (P25, P75)	5.96 (3.42, 11.48)
Male	74 (55.22)	<3	32 (23.88%)
		3–10	60 (44.78%)
WBC count (× 10^9^/L)		10–18	42 (31.34%)
P50 (P25, P75)	13.88 (5.55, 46.79)		
0–50	102 (76.12)	FAB classification	
50–100	18 (13.43)	M0	2 (1.49)
≥100	14 (10.45)	M1	5 (3.73)
		M2	68 (50.75)
CNSL		M4	14 (10.45)
CNSL1	123 (91.79)	M5	28 (20.90)
CNSL2	4 (2.99)	M6	1 (0.75)
CNSL3	7 (5.22)	M7	9 (6.72)
		NA	7 (5.22)
Chromosome karyotype			
normal	34 (25.37)	Number of fusions	
abnormal	89 (66.42)	Level 1	5 (7.46)
NA	11 (8.21)	Level 2	8 (11.94)
Structural chromosomal abnormalities		Level 3	54 (80.60)
t(8;21)(q22;q22)	30 (33.71)		
inv(16)(p13q22)	7 (7.87)	Number of mutations	
t(9;11)(p21;q23)	7 (7.87)	Level 1	16 (2.88)
t(6;9)(p23;q34)	1 (1.12)	Level 2	58 (10.45)
Other *	2 (2.25)	Level 3	481 (86.67)
Risk stratification at initial diagnosis		Final risk stratification	
LR	39 (29.10)	LR	14 (10.45)
IR	51 (38.06)	IR	27 (20.15)
HR	40 (29.85)	HR	69 (51.49)
NA	4 (2.99)	NA	24 (17.91)
HSCT			
YES	21 (15.67)	First event	
NO	113 (84.33)	Alive	66 (49.25)
FAB classification of transplanted patients		Relapse	22 (16.42)
M2	9 (42.86)	Death	9 (6.72)
M5	9 (42.86)	Failure of induction chemotherapy	12 (8.96)
M7	2 (9.52)	Therapy-Related Malignancy	1 (0.75)
NA	1 (4.76)	NA	24 (17.91)

Other *: t(1;7)(q21;q22),inv(3)(q21q26),del(11)(q14),+21,inc[cp3] and add(2)(q33),der(14;22)(q10;q10),+21[9]/idem,+14,der(14;22)(q10;q10),+22[1], respetivcly. WBC: White blood cell; CNSL: Central nervous system leukemia; HSCT: Hematopoietic Stem Cell Transplantation.

**Table 2:** Spectrum of Fusion Genes Identified in the Pediatric AML Cohort.

	Numbers	Age (Years), P50 (P25, P75)	Male	FAB (N)
LEVEL1				
*RUNX1::RUNX1T1*	32	6.96 (4.65, 11.77)	21	M2(32)
*KMT2A* rearrangement	29	3.5 (2.08, 7.42)	13	M2(5), M5(20), M4(3), NA (1)
*CBFB::MYH11*	13	10.33 (7.42, 12)	7	M2(2), M4(9), NA (2)
*DEK::NUP214*	1	5.67	0	M2(1)
*NUP98::KDM5A*	1	2.67	1	M7(1)
LEVEL2				
*CBFA2T3::GLIS2*	4	1.83 (1.39, 3.31)	2	M7(4)
*FUS::ERG*	4	3.21 (2.04, 6.48)	2	M5(3), M2(1)
*NUP98::NSD1*	2	3.38 (1.33, 5.42)	1	M2(1), M4(1)
*DDX3X::MLLT10*	2	7.25 (0.83, 13.67)	0	M1(1), M5(1)
*FUS::FEV*	1	3.75	1	M5(1)
*ZEB2::BCL11B*	1	8.08	1	M5(1)
*PICALM::MLLT10*	1	9.33	1	M0(1)
*TBC1D15::RAB21*	1	3.58	10	M2(1)

**Table 3:** Spectrum of Gene Mutations Identified in the Pediatric AML Cohort.

	Numbers	Age, P50 (P25, P75), /Years	Male	FAB (N)
LEVEL1				
*NRAS*	31	7.42 (2.67, 10.88)	15	M1(1), M2(15), M4(10), M5(2), M7(1), NA (2)
*FLT3*	25	9.42 (5.25, 12.33)	13	M1(2), M2(12), M4(5), M5(4), NA (2)
*FLT3*-ITD	12	7.58 (5.04, 11.98)	8	M1(2), M2(5), M4(1), M5(2), NA (2)
*FLT3*-TKD	14	9.13 (5.42, 12.33)	6	M2(7), M4(4), M5(3)
*KIT*	24	9.5 (5.73, 11.69)	18	M2(19), M4(5)
*KIT*-E17	16	9.5 (5.73, 12.35)	11	M2(9), M4(2)
*KIT*-E8	9	8.50 (3.83, 10.92)	6	M2(6), M4(3)
*CEBPA*	14	7.88 (5.54, 12.17)	6	M1(2), M2(12)
*CEBPA* ^bi^	13	8.08 (6.42,13.42)	5	M1(1), M2(12)
*CEBPA* ^sm^	1	5.25	1	M1(1)
*WT1*	13	7.42 (4.67, 11.58)	7	M1(2), M2(7), M4(1), M5(2), NA (1)
*KRAS*	11	2.58 (1.83, 8.63)	8	M2(3), M4(2), M5(5),NA(1)
*PTPN11*	7	5.42 (2.83, 9.08)	7	M0(1), M2(2), M5(3), M6(1)
*NPM1*	3	9.67 (6.25, 11.79)	0	M1(1), M2(2)
*RUNX1*	2	13.67 (12.58, 14.75)	1	M2(1), NA (1)
*U2AF1*	2	6.38 (3.42, 9.33)	1	M5(1), M0(1)
*ASXL1*	2	3.96 (1.67, 6.25)	2	M5(2)
*TP53*	2	11.09 (6.25, 15.92)	2	M5(1), NA (1)
*IDH2*	1	9.67	0	M1(1)
*KMT2A*	1	14.75	0	M2(1)
*SRSF2*	1	9.83	1	M2(1)
*NF1*	1	12.75	1	M0(1)
LEVEL2				
*ASXL2*	10	5.25 (4.35, 9.54)	5	M2(10)
*CSF3R*	7	12.67 (7, 12.83)	3	M2(7)
*CEBPA*	6	11.46 (4.56, 13.23)	1	M1(2), M2(1), NA (3)
*GATA2*	5	8.08 (7.67, 13.92)	1	M2(4), M5(1)
*EP300*	4	13.33 (11.65, 14.35)	3	M2(2), M4(1), NA (1)
*RAD21*	4	4.38 (2.85, 6.65)	3	M2(3), M7(1)
*CTCF*	4	4.17 (3.75, 6.54)	3	M2(2), M5(2)
*ETV6*	4	3.42 (2.96, 4.17)	3	M2(2), M5(1), M6(1)
*KDM6A*	4	6.71 (4.83, 9.48)	3	M5(2), M2(2)
*FLT3*	4	8.29 (3.21, 12.90)	1	M5(3), NA (1)
*SETD2*	3	3.42 (3.08 5.42)	0	M5(3)
*MYC*	3	8.42 (5.25, 9.13)	2	M2(1), M4(1), M7(1)
*PHF6*	3	13.67 (10.04, 14.58)	1	M1(1), M2(2)
*EZH2*	3	7.42 (6.67, 9.92)	2	M2(3)
*NF1*	3	12.33 (10.58, 14.13)	3	M4(2), NA2(1)
*DXH15*	2	8 (3.67, 12.33)	1	M2(2)
*IKZF1*	2	7.67 (2.58, 12.75)	1	M0(1), M2(1)
*JAK2*	2	7.67 (0.92, 14.42)	2	M2(1), M7(1)
*JAK3*	2	3.34 (2.42, 4.25)	0	M2(1), NA (1)
*DNM2*	2	8.25 (3.83, 12.67)	1	M2(2)
*CBL*	2	8.17 (5.25, 11.08)	2	M2(2)
*UBTF*	2	12.25(11.17, 13.33)	2	M2(2)
*PTEN*	2	8.96 (3.83, 14.08)	2	M2(2)
*CCND2*	2	7.08 (3.83, 10.33)	2	M2(2)
*KMT2D*	2	12.05 (11.67, 12.42)	1	M2(1), M5(1)
*KMT2C*	2	10.59 (6.42, 14.75)	1	M2(2)
*MGA*	2	5.79 (5.67, 5.92)	0	M2(2)
*BRCA2*	2	5.54 (5.42, 5.67)	1	M2(1), M6(1)
*MN1*	1	1.25	1	M2(1)
*TET2*	1	1.25	1	M2(1)
*ATM*	1	8.42	0	M2(1)
*MAP3K1*	1	3.67	0	M2(1)
*NSD1*	1	4.25	0	M2(1)
*NOTCH2*	1	8.83	1	M4(1)
*ARID2*	1	2.75	1	M4(1)
*XPC*	1	12.75	1	M0(1)
*DIS3*	1	3.67	0	M2(1)
*DNMT3A*	1	3.58	0	M5(1)
*EED*	1	3.75	1	M5(1)
*BRAF*	1	3.83	1	M2(1)
*BUB1B*	1	2.58	1	M4(1)
*MED12*	1	2.08	1	M4(1)
*MSH6*	1	8.33	0	M1(1)
*NFE2*	1	3.50	0	M2(1)
*ATRX*	1	10.08	0	NA (1)
*FBOX11*	1	5.42	1	M2(1)
*GATA1*	1	2.17	1	M7(1)
*MAX*	1	9.83	1	M4(1)
*PCBP1*	1	10.33	1	M2(1)
*STAG2*	1	5.25	1	M2(1)
*SMC1A*	1	4.33	1	M5(1)
*XRCC2*	1	8.33	0	M1(1)
*U2AF1*	1	12.42	1	M2(1)
*PTPN11*	1	9.33	1	M0(1)
*SRSF2*	1	14.75	0	M2(1)
*TP53*	1	2.08	0	M2(1)
*WT1*	1	9.33	1	M0(1)
*KIT*	1	5.25	1	M2(1)

*FLT3*-ITD: *FLT3* internal tandem duplication, *FLT3*-TKD: Tyrosine kinase domain (amino acids 835 and 836) of *FLT3*, *KIT*-E17: *KIT* exon 17, *KIT*-E8: *KIT* exon 8, *CEBPA*^bi^: Biallelic mutations of the *CEBPA* gene, *CEBPA*^sm^: Monoallelic mutation of the *CEBPA* gene.

## Data Availability

The data presented in this study are available on request from the corresponding author. The data are not publicly available due to privacy and confidentiality considerations.

## Supplementary Materials

The following supporting information can be downloaded at: https://www.mdpi.com/article/10.3390/curroncol32110605/s1, Figure S1: (A) Risk stratification criteria. (B)The treatment protocol of 2015 AML03. Bone marrow evaluation: assessment of complete remission by bone marrow morphology. The ADE (10+3+5) regimen was given in the first course, including Cytarabine (AraC) 100 mg/m^2^/dose every 12 h on days 1–10, Daunorubicin (DNR) 50 mg/m^2^ once daily on day 1, 3, 5, and Etoposide (VP-16, E) 100 mg/m^2^ every day on days 1–5. During the second course of induction therapy, patients in low-risk group received ADE (8+3+5) regimen, including AraC 100 mg/m^2^/dose every 12 h on days 1–8, DNR 50 mg/m^2^ once daily on days 1, 3, 5, and VP-16 100 mg/m^2^ every day on days 1–5. Patients in intermediate or high-risk groups received an AM regimen, including AraC 1 g/m^2^/dose every 12 h on days 1–4, Mitoxantrone 12 mg/m^2^ once daily on days 3–6. During the first course of intensification therapy, patients received AraC 1 g/m^2^/dose every 12 h on days 1–5 and VP-16 150 mg/m^2^ once daily on days 1–5. During the second course of intensification therapy, patients in the low-risk group received an AM regimen, including AraC 1 g/m^2^/dose every 12 h on days 1–4, and Mitoxantrone 12 mg/m^2^ once daily on days 3–6. Patients in intermediate or high-risk groups received an AL regimen, including AraC 3 g/m^2^/dose every 12 h on days 1, 2, 8, 9, and L-asparaginase (L-asp) 6000 U/m^2^ once daily on days 2 (42 h after 1st AraC dose on day 1) and day 9 (42 h after 1st AraC dose on day 8); Figure S2: (A) Kaplan–Meier curves of event-free survival of patients with and without *PTPN11* mutation. (B) Kaplan–Meier curves of event-free survival of patients with and without KRAS mutation. (C) Kaplan–Meier curves of event-free survival of patients with and without *CEBPA* mutation. (D) Kaplan–Meier curves of event-free survival of patients with and without *NRAS* mutation. (E) Kaplan–Meier curves of event-free survival of patients with and without *KIT* mutation. (F) Kaplan–Meier curves of event-free survival of patients with and without *KIT*-E8 mutation. (G) Kaplan–Meiercurves of event-free survival of patients with and without *KIT*-E17 mutation. (H) Kaplan–Meier curves of event-free survival of patients with and without *FLT3*-TKD mutation; Table S1: Classification criteria for Level 1–3 fusion genes and mutated genes; Table S2: Gene Mutations and Fusions in pAML Patients Who Received HSCT; Table S3: Fusions identified from RNA-seq; Table S4: Pairwise association between mutations; Table S5: Uni- and multivariable Cox analysis of variables impacting EFS; Table S6: Uni- and multivariable Logistic regression analysis of variables impacting CR; Table S7: Novel Fusions identified from RNA-seq.
