# Supplementary material for: Unveiling the Genetic Mosaic of Pediatric AML: Insights from Southwest China
Source: Curr Oncol. 2025 Oct 30;32(11):605. doi: 10.3390/curroncol32110605 (PMC12651160; doi:10.3390/curroncol32110605)
Supplement: Supplementary file 1 [file curroncol-32-00605-s001.zip › Supplementary Table 1 .pdf]

Supplementary Table 1: Classification criteria for Level 1-3 fusion genes and mutated genes

Levels of evidence	Category	Therapeutic	Diagnosis	Prognosis
**Level 1**	**Level A**	1. Biomarkers that predict response or Resistance to FDA-approved therapies for a specific type of tumor. 2. Biomarkers included in professional guidelines that predict response or resistance to therapies for a specific type of tumor.	Biomarkers included in professional guidelines as diagnostic for a specific type of tumor.	Biomarkers included in professional guidelines as prognostic for a specific type of tumor.
	**Level B**	Biomarkers that predict response or resistance to therapies for a specific type of tumor based on well-powered studies with consensus from experts in the field.	Biomarkers of diagnostic significance for a specific type of tumor based on well-powered studies with consensus from experts in the field.	Biomarkers of prognostic significance for a specific type of tumor based on well-powered studies with consensus from experts in the field.
**Level 2**	**Level C**	1. Biomarkers that predict response or resistance to therapies approved by the FDA or professional societies for a different type of tumor. 2. Biomarkers that serve as inclusion criteria for clinical trials.	Biomarkers of diagnostic significance based on the results of multiple small studies.	Biomarkers of prognostic significance based on the results of multiple small Studies.
	**Level D**	Biomarkers that show plausible therapeutic significance based on preclinical studies.	Biomarkers that may assist disease diagnosis themselves or along with other biomarkers based on small studies or a few case reports.	Biomarkers that may assist disease prognosis itself or along with other biomarkers based on small studies or a few case reports.
**Level 3**	/	Biomarkers with unclear clinical implications, such as biomarkers exhibiting low population frequency (rare in general populations).	Biomarkers may serve as potential clues suggesting associations between certain biomarkers and a disease, but they lack sufficient evidence to support definitive diagnostic value.	Biomarkers may suggest potential risks or unidentified factors in prognosis, but they fail to quantify their impact on survival or recurrence rates.
