# Supplementary material for: Unveiling the Genetic Mosaic of Pediatric AML: Insights from Southwest China
Source: Curr Oncol. 2025 Oct 30;32(11):605. doi: 10.3390/curroncol32110605 (PMC12651160; doi:10.3390/curroncol32110605)
Supplement: Supplementary file 1 [file curroncol-32-00605-s001.zip › Supplementary Table 2.pdf]

Supplementary Table 2: Gene Mutations and Fusions in pAML Patients Who Received HSCT

Level 1 gene fusions	Level 1 gene mutations
KMT2A rearrangement (7, M2_1/7, M5_6/7)	*ASXL1* (1, M5_1/1)
NUP98-KDM5A (1, M7_1/1)	*CEBPA* (1, M2_1/1)
RUNX1-RUNX1T1 (5, M2_5/5)	*FLT3* (4, M2_2/4, M5_1/4, NA_1/4)
	FLT3-ITD (3, M2_1/3, M5_1/3, NA_1/3)
	FLT3-TKD (2, M2_1/2, M5_1/2)
	KIT (3, M2_3/3)
	KIT-E17(3, M2_3/3)
	KRAS (3, M2_1/3, M5_2/3)
	NRAS (3, M2_1/3, M5_1/3, NA_1/3)
	PTPN11 (2, M5_2/2)
	WT1 (5, M2_3/5, M5_1/5, NA_1/5)
