# Supplementary material for: Unveiling the Genetic Mosaic of Pediatric AML: Insights from Southwest China
Source: Curr Oncol. 2025 Oct 30;32(11):605. doi: 10.3390/curroncol32110605 (PMC12651160; doi:10.3390/curroncol32110605)
Supplement: Supplementary file 1 [file curroncol-32-00605-s001.zip › Supplementary Table 5.pdf]

Supplementary Table 5: Uni- and multivariable Cox analysis of variables impacting EFS

Variable	Groups	EFS			
		Univariable analysis		Multivariable analysis	
		HR (95% CI)	*P*	HR (95% CI)	*P*
Gender	Female vs. Male	0.956 (0.525-1.741)	0.882		
Age at diagnosis (y)	Continuous, years	0.780 (0.384-1.584)	0.492		
WBC at diagnosis (×10^9/L)	Continuous	1.002 (0.998 - 1.006)	0.368		
ASXL1	Yes vs. No	3.660 (0.870-15.398)	0.077	4.184 (0.985-17.774)	0.052
CEBPA	Yes vs. No	0.519 (0.160 - 1.679)	0.274		
FLT3	Yes vs. No	1.566 (0.789 - 3.110)	0.200		
FLT3-ITD	Yes vs. No	1.296 (0.510 - 3.295)	0.586		
FLT3-TKD	Yes vs. No	1.985 (0.883 - 4.464)	0.097		
KIT	Yes vs. No	1.224 (0.601 - 2.491)	0.577		
KIT-E17	Yes vs. No	0.952 (0.401 - 2.262)	0.912		
KIT-E8	Yes vs. No	1.422 (0.507 - 3.991)	0.504		
KRAS	Yes vs. No	2.043 (0.858 - 4.863)	0.106		
NRAS	Yes vs. No	1.350 (0.677 - 2.691)	0.394		
PTPN11	Yes vs. No	0.371 (0.051 - 2.700)	0.327		
RUNX1	Yes vs. No	1.491 (0.204 - 10.879)	0.694		
TP53	Yes vs. No	5.183 (0.690 - 38.958)	0.110		
WT1	Yes vs. No	2.285 (1.053 - 4.956)	0.037	2.400 (1.101-5.233)	0.028
CBFB::MYH11	Yes vs. No	1.445 (0.609 - 3.428)	0.403		
RUNX1::RUNX1T1	Yes vs. No	0.443 (0.197 - 0.998)	0.049		
KMT2Ar	Yes vs. No	1.445 (0.711 - 2.939)	0.309		

Abbreviations: CI, confidence interval; HR, hazard ratio; WBC, white blood cell; EFS: event-free survival; *KMT2Ar, KMT2A* rearrangement.
