# Supplementary material for: Unveiling the Genetic Mosaic of Pediatric AML: Insights from Southwest China
Source: Curr Oncol. 2025 Oct 30;32(11):605. doi: 10.3390/curroncol32110605 (PMC12651160; doi:10.3390/curroncol32110605)
Supplement: Supplementary file 1 [file curroncol-32-00605-s001.zip › Supplementary Table 6.pdf]

Supplementary Table 6: Uni- and multivariable Logistic regression analysis of variables impacting CR

Variable	Groups	CR			
		Univariable analysis		Multivariable analysis	
		OR (95% CI)	*P*	OR (95% CI)	*P*
Gender	Female vs. Male	0.651 (0.171-2.467)	0.529		
Age at diagnosis (y)	Continuous, years	0.961 (0.828 -1.114)	0.595		
WBC at diagnosis ($\times 10^9$/L)	Continuous	0.989 (0.966 - 1.012)	0.347		
ASXL1	Yes vs. No	0 (0-inf)	0.999		
CEBPA	Yes vs. No	0 (0-inf)	0.999		
FLT3-ITD	Yes vs. No	10.400 (2.196 - 49.247)	0.003	10.400 (2.196 - 49.247)	0.003
FLT3-TKD	Yes vs. No	3.208 (0.553-18.614)	0.194		
KIT	Yes vs. No	0 (0-inf)	0.998		
KIT-E8	Yes vs. No	0 (0-inf)	0.999		
KIT-E17	Yes vs. No	0 (0-inf)	0.999		
KRAS	Yes vs. No	1.206 (0.133-10.956)	0.868		
NRAS	Yes vs. No	3.368 (0.881-12.879)	0.076		
PTPN11	Yes vs. No	0 (0-inf)	0.999		
TP53	Yes vs. No	0 (0-inf)	1		
WT1	Yes vs. No	3.524 (0.771 - 16.099)	0.104		
CBFB::MYH11	Yes vs. No	0 (0-inf)	0.999		
RUNX1::RUNX1T1	Yes vs. No	0.615 (0.122 - 3.107)	0.556		
KMT2Ar	Yes vs. No	1.133 (0.218 - 5.885)	0.882		

Abbreviations: CI, confidence interval; OR, odds ratio; WBC, white blood cell; CR: Complete remission; *KMT2Ar*, *KMT2A* rearrangement.
