# Supplementary figures and images for: Unveiling the Genetic Mosaic of Pediatric AML: Insights from Southwest China
Source: Curr Oncol. 2025 Oct 30;32(11):605. doi: 10.3390/curroncol32110605 (PMC12651160; doi:10.3390/curroncol32110605)

A

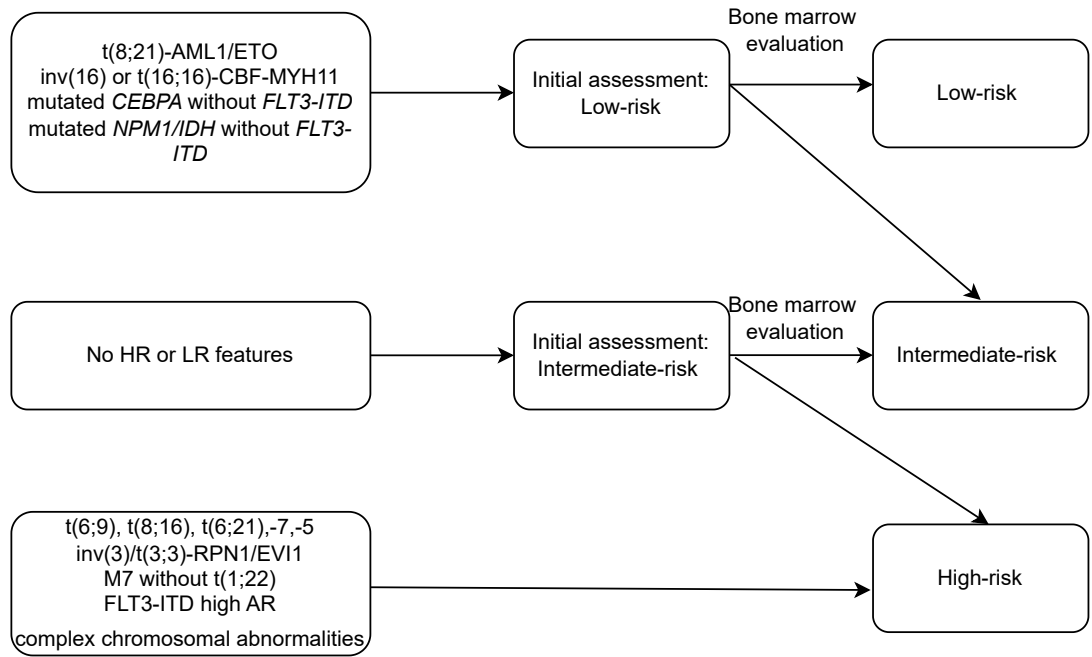

B

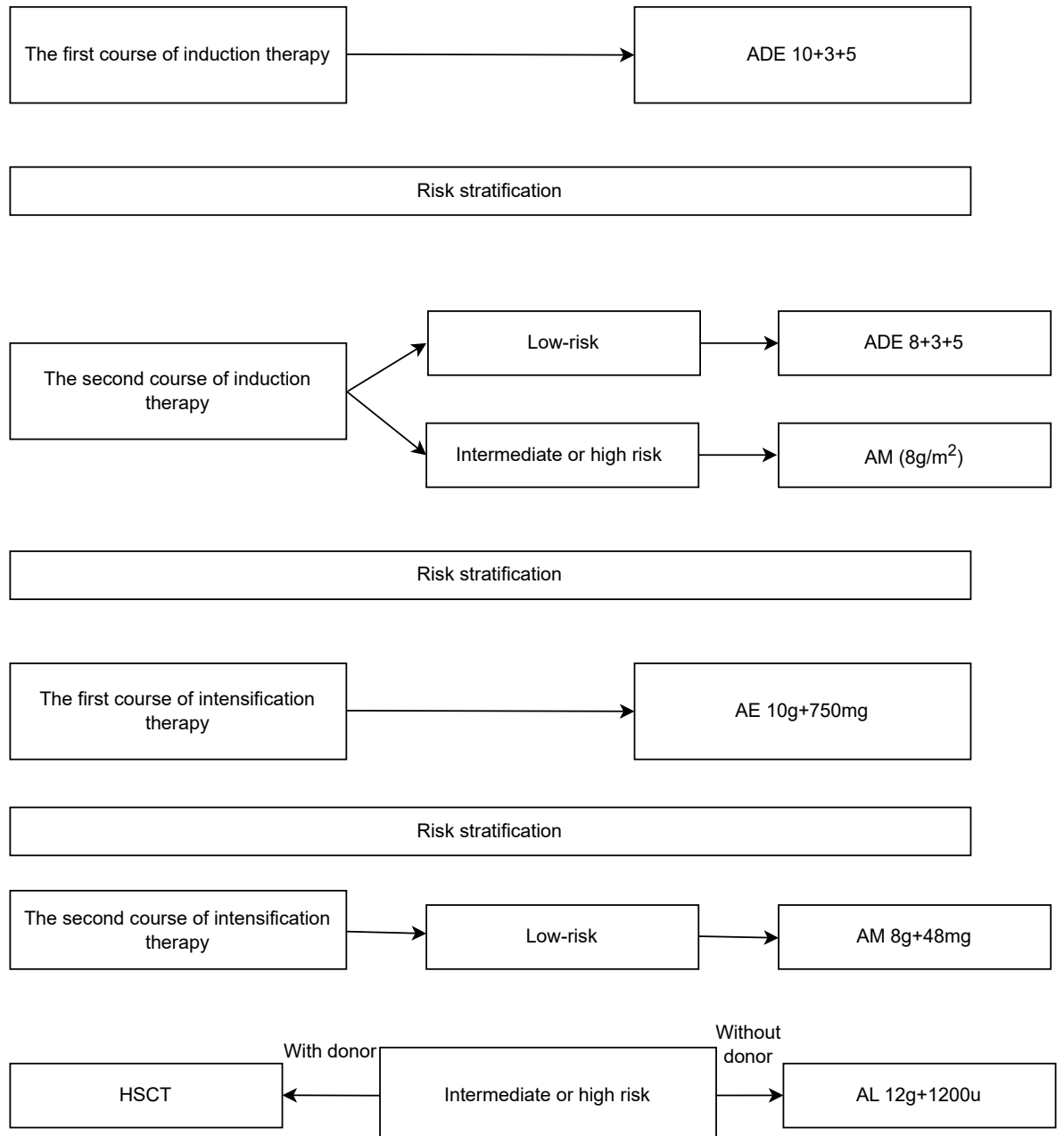

Supplement: Supplementary file 1 [file curroncol-32-00605-s001.zip › Supplementary Figure 1.pdf]

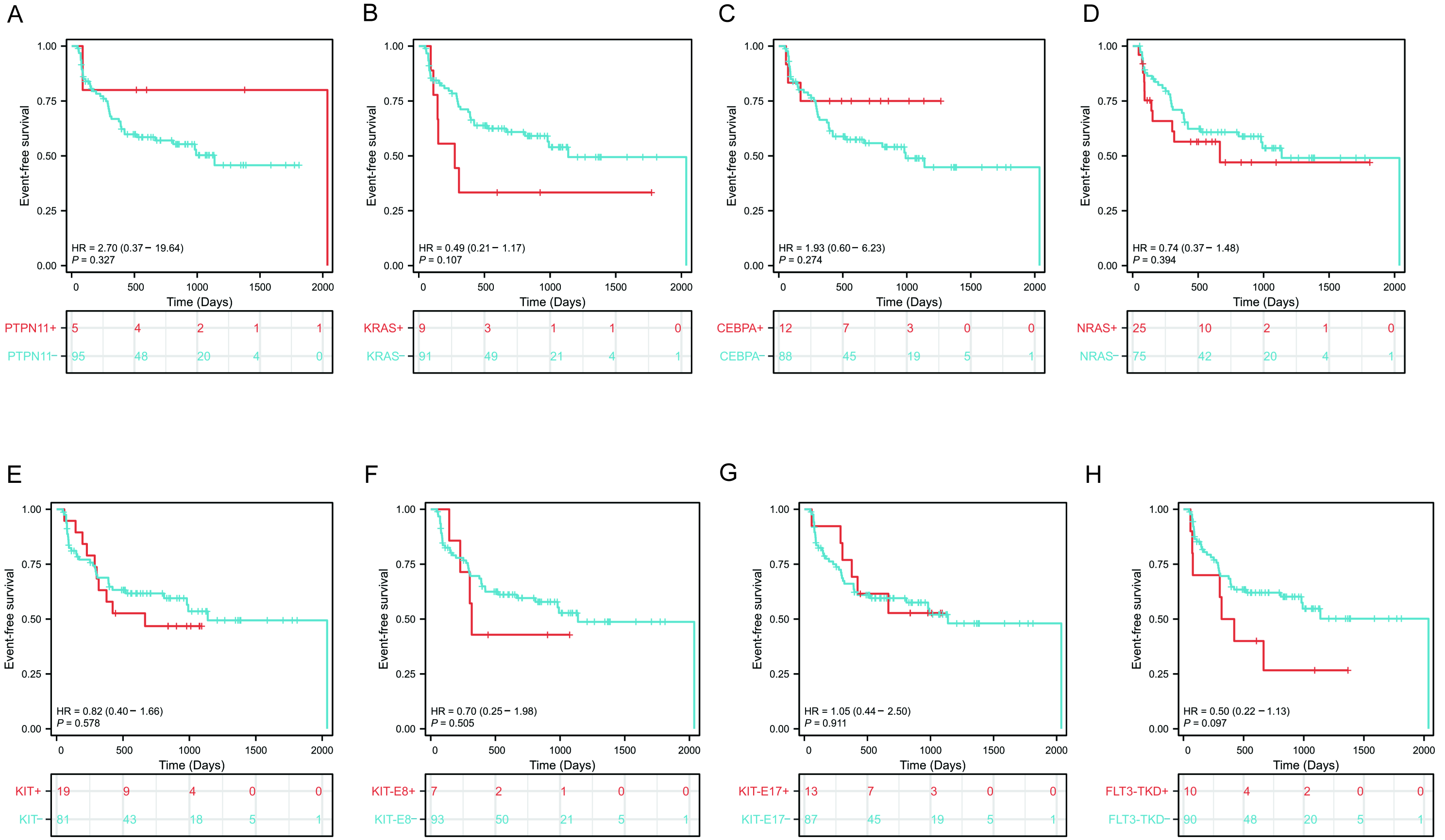

Supplement: Supplementary file 1 [file curroncol-32-00605-s001.zip › Supplementary Figure 2.tif]
